# Aluminium Injection Mould Behaviour Using Additive Manufacturing and Surface Engineering

**DOI:** 10.3390/ma18174216

**Published:** 2025-09-08

**Authors:** Marcelo José de Lima, Jorge Luis Braz Medeiros, José de Souza, Carlos Otávio Damas Martins, Luciano Volcanoglo Biehl

**Affiliations:** 1 Campus Carreiros, Federal University of Rio Grande (FURG), Av. Itália, Km 8, Rio Grande 96203-900, RS, Brazil; marcelo@mjlprojetos.com.br (M.J.d.L.); josesouza@liberato.com.br (J.d.S.); lucianobiehl@furg.br (L.V.B.); 2 Cidade Universitária Professor José Aloísio de Campos, Federal University of Sergipe (UFS), Avenida Marechal Rondon, s/n, Jardim Rosa Elze, São Cristovão 49100-000, SE, Brazil; cmartins@academico.ufs.br

**Keywords:** additive manufacturing, aluminium injection moulds, formed channels

## Abstract

This study evaluates the application of metal additive manufacturing—specifically the laser powder bed fusion (LPBF) process—for producing aluminium die-casting mould components, comparing 300-grade maraging steel inserts with conventional H13 tool steel. Efficient thermal management and mould durability are critical in aluminium injection moulding. Still, traditional machining limits the design of cooling channels, resulting in hot spots, accelerated wear, and a reduced service life. LPBF allows the fabrication of complex geometries, enabling conformal cooling channels to enhance thermal control. Component samples were manufactured using maraging steel via LPBF, machined to final dimensions, and subjected to duplex surface treatment (plasma nitriding + CrAlN PVD coating). Thermal performance, dimensional stability, mechanical properties, and wear resistance were experimentally assessed under conditions simulating industrial production. The results demonstrate that LPBF components with optimised cooling channels and surface engineering achieve higher thermal efficiency, an extended service life (up to 2.6×), improved hardness profiles (545 HV0.05 core, 1230 HV0.05 on nitrided surface and 2850 HV0.05 after PVD film deposition), and reduced maintenance frequencies compared to H13 inserts. The study confirms that additive manufacturing, combined with tailored surface treatments and optimised cooling design, overcomes the geometric and thermal limitations of conventional manufacturing, offering a reliable and productive solution for aluminium die-casting moulds.

## 1. Introduction

The aluminium die-casting industry has historically faced significant challenges related to the durability and operational efficiency of dies, as well as the costs associated with their manufacture and maintenance [1,2,3,4,5]. Among the main problems observed are the accelerated wear of components, the formation of thermal cracks, and structural failures—factors that compromise not only the integrity and quality of moulded parts but also the economic viability of projects and the overall productivity of operations [5,6,7,8]. These limitations are exacerbated by the restrictions inherent in conventional manufacturing processes, especially with regard to the design of the cooling circuits [9,10,11,12]. The limited geometry of the cooling channels obtained by traditional machining makes it difficult to standardise thermal dissipation, resulting in the formation of critical heating zones. This is widely recognised as one of the main factors responsible for reducing the useful life of moulds and reducing the efficiency of the injection process [12,13,14]. In this context, metal additive manufacturing (MAM), particularly the laser powder bed fusion (LPBF) process, has emerged as a disruptive technological alternative [2,15,16]. Some studies have explored the application of alternative beam shapes in LPBF to address the existing limitations due to the Gaussian beam shape [2,11,17]. Differentiated beam shapes seek to reduce the disadvantages of the Gaussian beam shape and improve the performance of LPBF processes [15,17]. By modifying the intensity of the laser beam or the heat profile, new process conditions can be achieved. In the LPBF process, the metal powder is dispersed in the delivery system by a coating blade, while the build platform, mounted on a piston, is lowered to a height of the same value as the thickness of the slice to be melted. This condition is repeated layer after layer until the final piece is obtained. The LPBF process takes place with a continuous flow and an inert gas atmosphere (argon or nitrogen) [15,17]. The ability to manufacture components layer by layer enables the creation of complex, internalised geometries, such as conformal cooling channels, which would not be feasible using conventional processes [15,16]. This solution enables significantly more efficient thermal control, reducing temperature gradients and minimising the occurrence of thermal failures. The incorporation of additive manufacturing in the production of mould components, in addition to optimising thermal performance, results in additional benefits, such as increased durability of the moulds and a consequent reduction in operating costs throughout their life cycle. In this way, MAM presents itself as a technology capable of transforming the current paradigm in the aluminium injection mould industry, promoting advances in both reliability and productivity. Aluminium die casting is a production process that consists of injecting molten aluminium under pressure and at high speeds into a metal mould in order to produce parts in series [15,16]. The main advantage of production with the aluminium pressure injection process is the ability to manufacture large quantities of parts with high quality, repeatability, and dimensional control [17]. However, the aluminium pressure injection process, unlike plastic injection processes, has considerably larger thermomechanical stresses, which make it difficult to control and ensure the accuracy of the process parameters throughout production batches [17,18]. Additive manufacturing, also known as 3D printing, has established itself as a disruptive technology in the mould industry, especially in the production of components for aluminium injection moulds under pressure [19,20]. Its ability to manufacture complex geometries that are difficult to execute using conventional methods—often unfeasible or economically uncompetitive—makes this technology a promising solution to critical engineering challenges. The physical vapour deposition (PVD) process is a surface engineering technique that improves the tribological properties of steels. It is used in moulds, cutting tools, and components subjected to wear [5]. This adhesive deposition of thin films results in high surface microhardness, and, due to the difference in microhardness compared to the core, chipping can occur [5]. The use of duplex treatment (nitriding and PVD) increases the surface microhardness, thus improving the anchoring capacity of the PVD coating and reducing the risk of debonding, a phenomenon characterised by the fragile failure of the coating due to insufficient substrate support [5,6,7]. Plasma nitriding is usually the most widely used in the duplex process, with the best range of temperature options and control of the diffusion layer and compound layer. However, there are applications with gaseous nitriding with the greater depth of the nitrided layer [6,7]. In plasma nitriding, molecular nitrogen in gas form is ionised in a glowing plasma discharge, generating ions that diffuse from the surface. In gaseous nitriding, nitrogen is generated from the dissociation of ammonia, with a more restricted heat treatment range [6]. Maraging steel is characterised as a low-carbon martensitic grade containing large amounts of nickel and cobalt as the main alloying elements [9,10]. This steel is characterised by antagonistic mechanical properties, such as ultra-high strength and high fracture toughness [9,10]. The main hardening phenomena in these alloys are related to precipitates such as Fe_2_Mo, Ni_3_Mo, and Fe_7_Mo_6_. Due to their low carbon content, these steels are highly weldable. In addition, the low carbon content of maraging steel provides excellent weldability [9,10]. This study seeks to address the gaps in the manufacturing process of components applied to aluminium injection, indicating the main process parameters. The metallurgical and mechanical properties of the manufacturing process are also verified [21,22,23,24,25,26,27,28,29,30,31,32,33,34,35,36].

## 2. Materials and Methods

### 2.1. Materials and Manufacturing Process

The component samples were fabricated from DIN 1.2709 maraging steel using the laser powder bed fusion (LPBF) process in a TruPrint 3000 system (TRUMPF Laser GmbH, Ditzingen, Germany). A 3 mm machining allowance was added to all surfaces to ensure dimensional accuracy and allow subsequent finishing operations.

### 2.2. Post-Processing and Machining

After fabrication, the components underwent finish machining in a HERMLE 5-axis machining centre, complemented by wire EDM (Agie Charmilles Form 3000, Georg Fischer AG, Losone, Switzerland). Squaring and drilling of reference holes were performed to allow precise positioning in the EROWA modular system. Dimensional inspection was carried out to verify conformity with design requirements. Final polishing, ensuring surface roughness of Ra ≤ 3.2 μm on the moulding surfaces, was performed prior to surface treatments.

### 2.3. Surface Treatments

A duplex treatment was applied, consisting of plasma nitriding at 450 °C followed by the physical vapour deposition (PVD) of a CrAlN coating. The coating thickness ranged from 6 to 12 μm.

### 2.4. Hardness and Microstructural Characterisation

Macrohardness measurements were performed using the Rockwell C (HR_C_) method, following the flat grinding of one surface to reduce the roughness. The microhardness profiles of the nitrided and coated layers were obtained by Vickers indentation at a 50 g load (HV_0.05_). Metallographic samples were prepared by standard procedures (sectioning, resin mounting, grinding and sand blasting at 1200 grit, polishing with diamond paste of 1 μm). Etching was performed with Kalling reagent, and microstructures were observed under an Olympus GX (Olympus, Tokyo, Japan)reflected light optical microscope.

### 2.5. Experimental Setup in Die Casting

The manufactured components were integrated into a die with two cavities: one equipped with the additively manufactured insert and the other with a conventional insert (H13 tool steel). Casting trials were performed on a 560 kN IDRA machine (IDRA, Travagliato, Itália), using deionised water at 65 °C and 6 bar as the cooling medium. After 100 continuous cycles, thermal stability was reached, and the surface temperatures were measured at predefined locations.

## 3. Results and Discussion

### 3.1. Design and Procurement of Components

**Figure 1:**
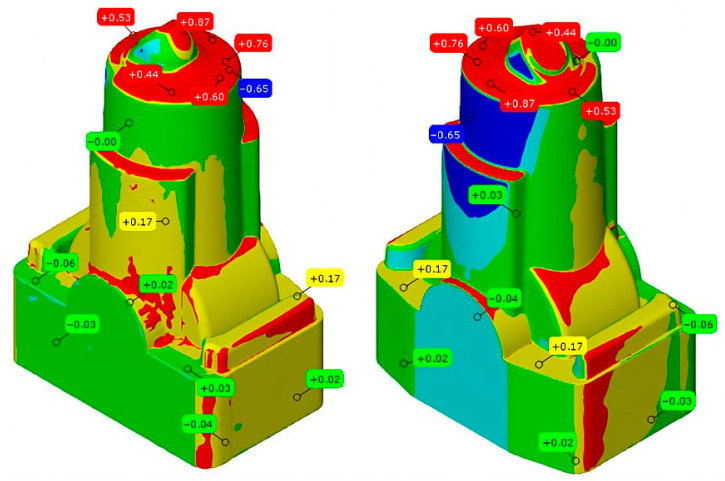
LPBF-printed component with dimensional deviation in the overmetal.

Figure 1 shows the design of the uncertain parts for aluminium injection. The decision was made to add 3 mm of overmetal to each side of the printed component due to dimensional deviations seen in preliminary tests [1,2,3]. The influence of contaminants in the metal powder during the setup process of the LPBF printers was verified, especially when changing materials between different production cycles. This residual contamination can significantly compromise the integrity of the printed substrate, affecting both its mechanical properties and its density [17].

The process began by clamping the component in a vice, allowing for squaring and the generation of initial references. Precision drilling was then carried out to fix it using EROWA system devices (EROWA AG, Männedorf, Switzerland), which were used from the second operation onwards, ensuring repeatability and dimensional accuracy in the subsequent machining stages.

**Figure 2:**
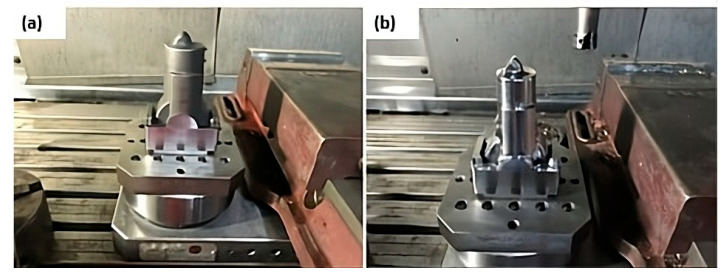
Component obtained by metal additive manufacturing (**a**) and in the machined condition to remove the overmetal (**b**).

Figure 2 shows the components obtained by metal additive manufacturing in (a) and machined from metal in (b). It should be noted that, in the manufacture of pressure injection moulds, machining usually represents the largest fraction of the production costs. Therefore, selecting a material with poor machinability can compromise the technical and economic viability of the project, making the prior analysis of this aspect essential for decision making [15,16,17].

**Figure 3:**
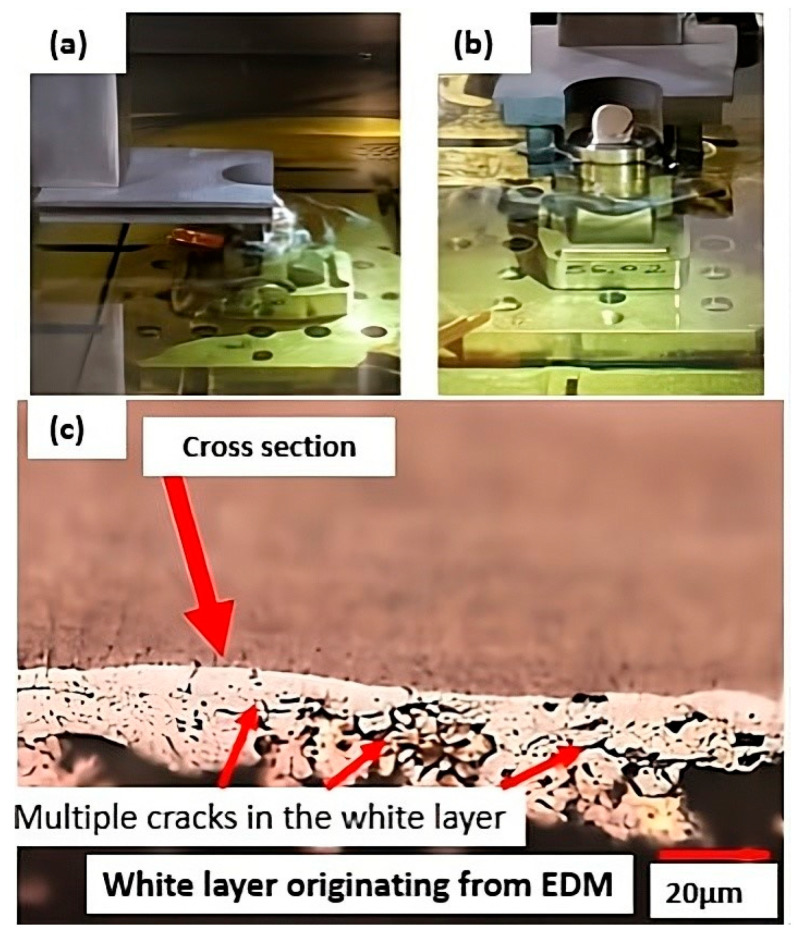
Part subjected to electroerosion in different directions in (**a**,**b**) and white layer generated with numerous microcracks (**c**).

Electrical discharge machining is an unconventional thermoelectric machining process that uses recurring faults to remove material from the workpiece [7,8]. There are still significant knowledge gaps regarding its application to steels obtained by additive manufacturing. The energy discharged per pulse and the combination of machining settings have a strong influence on the machining performance, affecting the material removal rate and surface roughness (Ra, Rt) and resulting in the formation of a white layer on top of the machining surface, which is deleterious [7,8]. Figure 3 shows the component after EDM, highlighting the white layer with numerous cracks. This layer was subsequently removed through manual sanding and polishing.

The presence of a white layer in the electrical discharge machining (EDM) process also tends to occur in both low-alloy and high-alloy steels. In tool steels used for cold work (e.g., D2, D6), hot work (e.g., H13, O1), and injection mould applications (e.g., P20, P40), the presence of this white layer can lead to significant embrittlement at edges and sharp corners, resulting in catastrophic failures [7,9]. In general, the greater the hardenability of the steel, the higher the tendency for embrittlement. In most alloy steels with high hardenability, embrittlement tends to be more intense than in maraging 300-grade steels, which harden by precipitation rather than by the distortion of the crystal lattice through martensitic transformation [8,11]. Dispersion in the dielectric is directly influenced by the powder concentrations in the EDM process, with a reduction in the number of particles having deleterious effects on the dielectric [7,8]. At higher concentrations, new conductive bridges are formed, which help to reduce electrode wear. However, at very high concentrations, particle agglomeration may occur, although the conductivity still tends to show highly favourable behaviour during the EWR process. Under these conditions, there is a tendency for the formation of more robust conductive bridges, which dissipate energy and, consequently, help to protect the electrodes [7,8]. This results in greater discharge stability, promoting more efficient energy dispersion in the EWR process.

### 3.2. Chemical and Microstructural Analysis

The chemical composition has a direct effect on the phase transformation mechanisms during hot electrical discharge machining (EDM). A small part of the molten cavity is ejected, with the crater formed by the spark representing only a proportion, i.e., 15% to 35% of the total volume of the molten material, while the rest of the material returns to the solid state, forming a resolidified layer, giving rise to thermal stresses. The presence of thermal stresses and overheating is extremely deleterious. The chemical composition, with the results shown in Table 1, was studied in order to minimise the effects of white layer formation. At the same time, a small portion of the vaporised material from the part remains close to the surface of the spark cavity, and, when the spark is turned off, it reconnects as a layer to the surface of the spark cavity. The layer is known as the redeposited layer, since the material has been separated from the surface of the part and then returned. Resolidified and redeposited layers are often called the white layer. Large variations in temperature and pressure during machining are the main factors that cause surface cracks, reducing the material’s resistance to fatigue and corrosion. Generally, the surface crack density increases with increasing pulse energy, although the crack mechanisms are much more complex [17].

**Figure 4:**
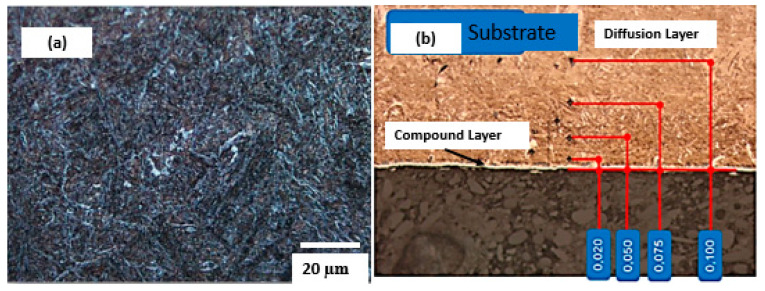
Substrate microstructure (**a**) and composite and diffusion layer of maraging steel (**b**).

The maraging steel used in metal additive manufacturing in this research is characterised as a high-alloy and low-carbon steel. They are practically carbon-free. After cooling in air from the austenitic field, they present martensite with a ductile and tenacious non-geminated morphology in the form of lath martensite. Its hardening mechanism is precipitation due to aging [18,19,20,21,22,23,24,25]. As the plasma nitriding and PVD deposition temperature was similar to the positioning temperature, the microstructure detected on the substrate was low-carbon martensite with the presence of coherent precipitates [18,19,20]. Precipitation hardening is caused by nanometre-sized intermetallic particles that precipitate during subsequent martensite aging (“maraging”) in the temperature range of 400 to 600 °C [9,10,22]. Figure 4 shows the microstructure of the maraging steel sample obtained by metal additive manufacturing. The martensite grain size has a direct influence on the mechanical properties. When there is spacing among the precipitated phases in the martensitic matrix with a smaller martensite grain size, the second phase helps to increase the mechanical strength [9,10].

It should be emphasised that the chemical compositions of both maraging 300-grade steel and hot work tool steel H13 contain nitride-forming elements that contribute to higher microhardness values, reducing the difference in microhardness between the PVD coating and the substrate [23,24,25,26,27]. This factor is extremely favourable for the anchoring of the deposited film, reducing chipping at the surface. The main alloying elements in maraging 300-grade steel that favour nitriding are Al, V, Mo, and Ti [23,24,25,26,27].

The CrAlN PVD deposit used was 6 µm. The advantage of using the duplex process (nitriding and PVD) is that the difference in the microhardness of the PVD thin film, followed by the diffusion compound layers from nitriding and the core microhardness, has a continuous profile, facilitating the anchoring of the PVD film and consequently reducing the possibility of detachment [24]. The presence of the diffusion layer and composite layer contributes to aspects other than anchoring the CrAlN thin film. The presence of the diffusion layer leads to an increase in compressive stresses, and the composite layer provides greater resistance to corrosion, considering that the PVD film applied does not attribute this condition to the substrate [24,34].

### 3.3. Surface and Core Microhardness Behaviour

The hardening mechanism of the substrates of components obtained by metal additive manufacturing was based on martensitic transformation, achieved by controlled cooling from the sintering temperature to the region, followed by homogenisation in the austenitic zone at a temperature of 850 °C. The samples were then cooled again to room temperature, generating a martensitic microstructure. The high Ni content of maraging steels inhibits the diffusion process that could generate the presence of ferrite and pearlite, resulting in low-carbon martensite [9,10,22]. This low-carbon martensite contributes to the toughness of the material. As the nitriding heat treatment and PVD thin film deposition occurred within the precipitation range of maraging steels (400 to 500 °C), the alloying elements precipitated, generating precipitates with Ni_3_Mo, Ni_3_Ti, NiAl, and Fe_2–3_Mo [9,10,22]. The high hardness of the substrate was accentuated by the presence of cobalt, which acted to decrease the solubility of Mo, favouring its precipitation. These precipitates anchor the movement of the dislocations, favouring an increase in the hardness of maraging steels [9,10,22].

**Figure 5:**
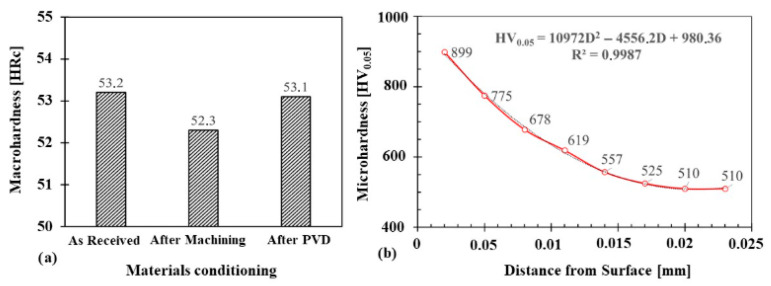
Microhardness profile of nitrided maraging steel with PVD film deposit. HRc hardness of the Maraging 300 steel substrate under different conditions (**a**), and microhardness profile after plasma nitriding (**b**).

The microhardness conditions of the substrate, diffusion layer, and composite layer (in plasma nitriding and PVD coating of AlCrN) are relevant for wear applications. Obtaining a smooth microhardness profile can contribute to increased fatigue life [26,27]. Even if the PVD thin film is not effective in terms of fatigue life and thermal fatigue, plasma nitriding contributes to the formation of compressive stresses, favouring the support of these phenomena [6,7,34]. Figure 5 shows the microhardness behaviour of the core, the nitrided layer, and the surfaces of the samples [30,31,32,33,34].

The polynomial regression (R^2^ = 0.9987) verified in the microhardness curve indicates a good correlation and homogeneous model, and the linear regression also presented a good and homogeneous model. The average hardness value found on the marbling steel substrate was 545 HV0.05, showing the aging condition during the plasma nitriding thermochemical cycle due to the similarity of the process temperatures [24,25,26,27,28,29,30,31,32,33,34,35]. The surface microhardness of the nitrided maraging 300-grade steel samples was 1230 HV0.05, and, after the application of the CrAlN thin film, it increased to 2850 HV0.05, exhibiting ceramic-like behaviour [34,35,36,37,38,39]. It should be noted that the diffusion process during nitriding, as well as the resulting microhardness, is associated with the presence of nitride-forming elements in the substrate [34,35,36,37,38,39]. In contrast, the hardness of the CrAlN thin film is independent of the substrate’s chemical composition. A good transition between the PVD coating and the substrate ensures better film adhesion and reduces the tendency for chipping [34,35,36,37,38,39]. Since thin films are only a few micrometres thick, nanoindentation testing is recommended to obtain more accurate measurements without penetrating the CrAlN layer. The hardness values of the substrates, measured using the HRC method, did not show significant variations before and after the application of the duplex treatment. The standard deviation of the hardness measurements was less than 1% in all cases, remaining within the tolerance limits of the measurement method [30,31,32,33,34,35,36,37,38,39,40].

### 3.4. Functional Test of Printed Insert

**Figure 6:**
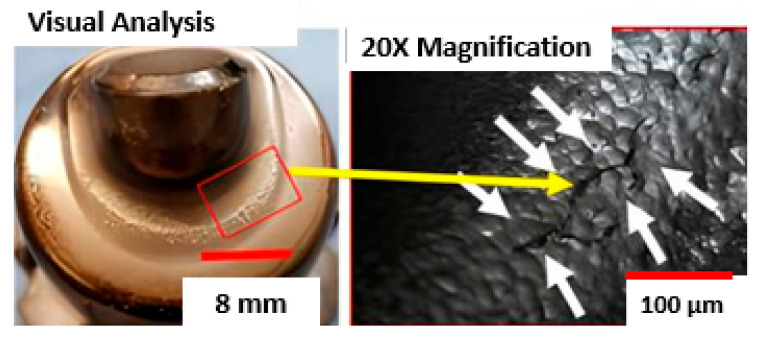
Surface erosion and thermal fatigue after 80,000 cycles.

The behaviour of the printed component, with the application of surface engineering, was evaluated in the aluminium pressure injection process. The thermal performance of the new cooling design with shaped channels was also evaluated. The optimised geometry of the channels led to a significant increase in the internal convection area, which resulted in better heat removal efficiency. During the test, it was found that the conformal cooling circuit promoted greater thermal uniformity and a higher heat dissipation rate compared to the conventional circuit. After the tests, the components were visually inspected, and it was observed that the additively manufactured component showed better dimensional stability. In addition, there was less residual aluminium adhesion on its surface when compared to the conventional component. This behaviour is directly related to the reduction in the operating temperature of the post with formed channels. It is important to note that, even with this thermal reduction, there was no loss in the formation of the release film. The surface temperature of the substrate remained within the ideal range to guarantee the formation and effective adhesion of the release agent [25,26,27,28,29,30,31,32,33,34,35,36,37]. Figure 6 shows the wear of the additively manufactured component after 80,000 cycles. The main degradation mechanisms found were thermal fatigue and surface erosion.

It has been found that the combination of a printed substrate and formed channels is highly effective for moulds used in aluminium die casting. This approach not only increases productivity and component life but also reduces the incidence of defects in the castings. Another crucial aspect identified was the reduction in the thermal gradient in the substrate, which promotes the more uniform distribution of thermal stresses during the production cycle [32,33,34,35,36,37]. This thermal homogeneity is one of the main factors in increasing the durability of the component. Based on the mathematical analysis of thermal stresses, it is understood that temperature variation in the substrate is one of the most determining factors in the occurrence of thermal fatigue [11,12]. In addition, the total absence of “pits” (microcavities) and oxides on the internal surfaces—phenomena frequently observed in series components—was noted. In conventional moulds, the combination of accentuated thermal variations, the chemical composition of the material, and the imperfections of the conventional drilling process favours the formation of heat concentration zones, which, when subjected to cyclical thermomechanical stresses, give rise to surface pits. These points act as thermal crack initiation nuclei, which, when associated with internal oxidation, accelerate the degradation of the component, leading to premature failure [11,12]. The results obtained reinforce the notion that additive manufacturing, by allowing the optimised shaping of channels and more homogeneous internal surfaces, is an effective solution to mitigate thermal degradation problems and substantially increase the useful life of pressure injection moulds. To summarise the main outcomes of this study, Table 2 presents the comparative performance of conventional H13 inserts and additively manufactured (MAM) 300-grade maraging steel inserts with conformal cooling channels. The table highlights the quantitative advantages of the AM components, including improved thermal performance, higher hardness, enhanced dimensional stability, and an extended service life. These results clearly demonstrate the benefits of combining metal additive manufacturing with surface engineering for aluminium die-casting industrial applications [38,39].

## 4. Conclusions

The metal additive manufacturing (MAM) of 300-grade maraging steel for aluminium die-casting mould components offers significant technical and competitive advantages over conventionally machined AISI H13 steel, particularly in applications requiring complex cooling geometries. Its relevance lies in the ability to produce shaped cooling channels that enhance thermal management, mould durability, and overall production efficiency. The main results indicate that components subjected to MAM with duplex surface treatment (plasma nitriding + CrAlN PVD coating) retained high substrate hardness (545 HV0.05) and surface microhardness (1230 HV0.05) after nitriding, enabling the effective anchoring of the CrAlN thin film, which exhibited microhardness of 2850 HV0.05. The additively manufactured inserts demonstrated dimensional stability and achieved up to a 2.6-times longer tool life compared to machined H13 inserts. Thermal performance was enhanced through the optimised cooling channel geometry, reducing thermal gradients and improving the quality of the injected parts. The machinability and EDM performance of 300-grade maraging steel were comparable to those of conventional tool steels, with only minor increases in electrode wear. Limitations of this study include the absence of an insert recovery analysis and evaluation of dimensional changes at the end of the service life. Future work will focus on the application of metal additive manufacturing in polymer injection moulds, the further optimisation of cooling channel design, and long-term industrial testing to maximise efficiency, durability, and reproducibility.

## Figures and Tables

**Table 1:** Chemical compositions of samples obtained by metal additive manufacturing.

Steel	%C	%Mn	%Si	%S	%P	%Co	%Mo	%Ni	%Ti	%Al	%V	%Cr
Maraging	0.017	0.054	0.088	0.057	0.019	9.29	4.74	18.5	0.85	0.114	0.10	0.02
H13	0.40	0.5	0.8	0.01	0.01	0.03	1.10	0.3	0.003	0.003	0.8	5.0

**Table 2:** Summary of main performance metrics comparing H13 and MAM 300-grade maraging steel inserts.

Parameter	H13 Steel Inserts	AM 300-Grade Maraging Steel Inserts	Improvements/Notes
Thermal uniformity	Moderate	High	Optimised conformal cooling channels reduce thermal gradients
Surface microhardness (HV_0.05_)	1100 with nitriding and 2780 after PVD	1230 with nitriding and 2850 after PVD	Significant increase due to duplex surface treatment
Dimensional stability	Good	Excellent	Reduced deformation under repeated cycles
Service life (cycles)	30,000	80,000	~2.6× longer
Residual aluminium adhesion	Moderate	Low	Improved surface finish and reduced temperature at the release agent interface

## Data Availability

The raw data supporting the conclusions of this article will be made available by the authors on request.
